# Thrombosed Middle Cerebral Artery Bifurcation Aneurysm Presenting as Acute Ischemic Stroke

**DOI:** 10.7759/cureus.85592

**Published:** 2025-06-09

**Authors:** Nicholas Dietz, Jasmine Omair, Vivien Dang, Kevin John, Brian J Williams, Isaac J Abecassis, Dale Ding

**Affiliations:** 1 Neurosurgery, University of Louisville, Louisville, USA; 2 Radiology, Stony Brook University, Stony Brook, USA

**Keywords:** ischemic stroke, m1 occlusion, mca aneurysm, parent artery occlusion, thrombosed aneurysm

## Abstract

Ischemic stroke caused by a thrombosed intracranial aneurysm is a rare clinical condition that presents distinct treatment challenges. We report the case of a 69-year-old woman with vascular comorbidities who was found unresponsive and presented with acute left-sided hemiplegia, aphasia, and rightward gaze deviation. Imaging revealed a large, unruptured, thrombosed aneurysm at the right middle cerebral artery bifurcation, with occlusion of the parent M1 segment. The patient underwent a right pterional craniotomy for resection of the thrombosed aneurysm and surgical clipping of three additional intracranial aneurysms. This case underscores the importance of including aneurysmal thrombosis in the differential diagnosis of ischemic stroke. Although management is complex, surgical resection and clipping of thrombosed aneurysms may offer a viable treatment approach.

## Introduction

Stroke is the second leading cause of death globally and ranks fifth in the United States, with a prevalence of 2.6% among US adults over the age of 20 [1]. Approximately 85% of all strokes are ischemic, and more than half of these occur within the middle cerebral artery (MCA) territory [2,3]. Although unruptured intracranial aneurysms are not typically linked to an increased risk of stroke, thrombosis within an aneurysm can, in rare cases, result in ischemic stroke [4]. Aneurysm size is a significant risk factor for spontaneous thrombosis, with 10-30% of large and giant aneurysms demonstrating partial thrombosis [5]. The most frequently affected sites for thrombosed aneurysms include the MCA (41%), the posterior communicating artery (15%), and the posterior inferior cerebellar artery (11%) [6].

To date, only nine cases have documented ischemic stroke secondary to a thrombosed aneurysm in the MCA territory [7-12]. Proposed mechanisms include migration of intraluminal thrombus into the parent artery, distal embolization, or vessel occlusion due to mechanical compression by the thrombosed mass. Management strategies vary from conservative observation to complex surgical interventions such as aneurysmal clipping, resection, and bypass procedures [13]. In this report, we present a case involving a large, unruptured, thrombosed right MCA bifurcation aneurysm that resulted in symptomatic ischemic stroke due to occlusion of the M1 segment. The patient successfully underwent craniotomy with aneurysm dome resection and surgical clipping without complication.

## Case presentation

In November 2024, a 69-year-old female with a history of cigarette smoking, hypertension, hyperlipidemia, coronary artery disease, irritable bowel syndrome, hypothyroidism, degenerative disc disease, and multinodular goiter presented to the emergency department after being found unresponsive at home. Her last known normal state was three days before presentation. On admission, she exhibited aphasia, left hemiplegia, left-sided facial droop, right gaze deviation, and impaired memory, with an initial National Institutes of Health Stroke Scale score of 22. Her home medications included diclofenac, hydrochlorothiazide-triamterene, atorvastatin, lisinopril, methimazole, and amlodipine. She also had a previously diagnosed pancreaticoduodenal artery aneurysm that had not been treated.

**Figure 1:**
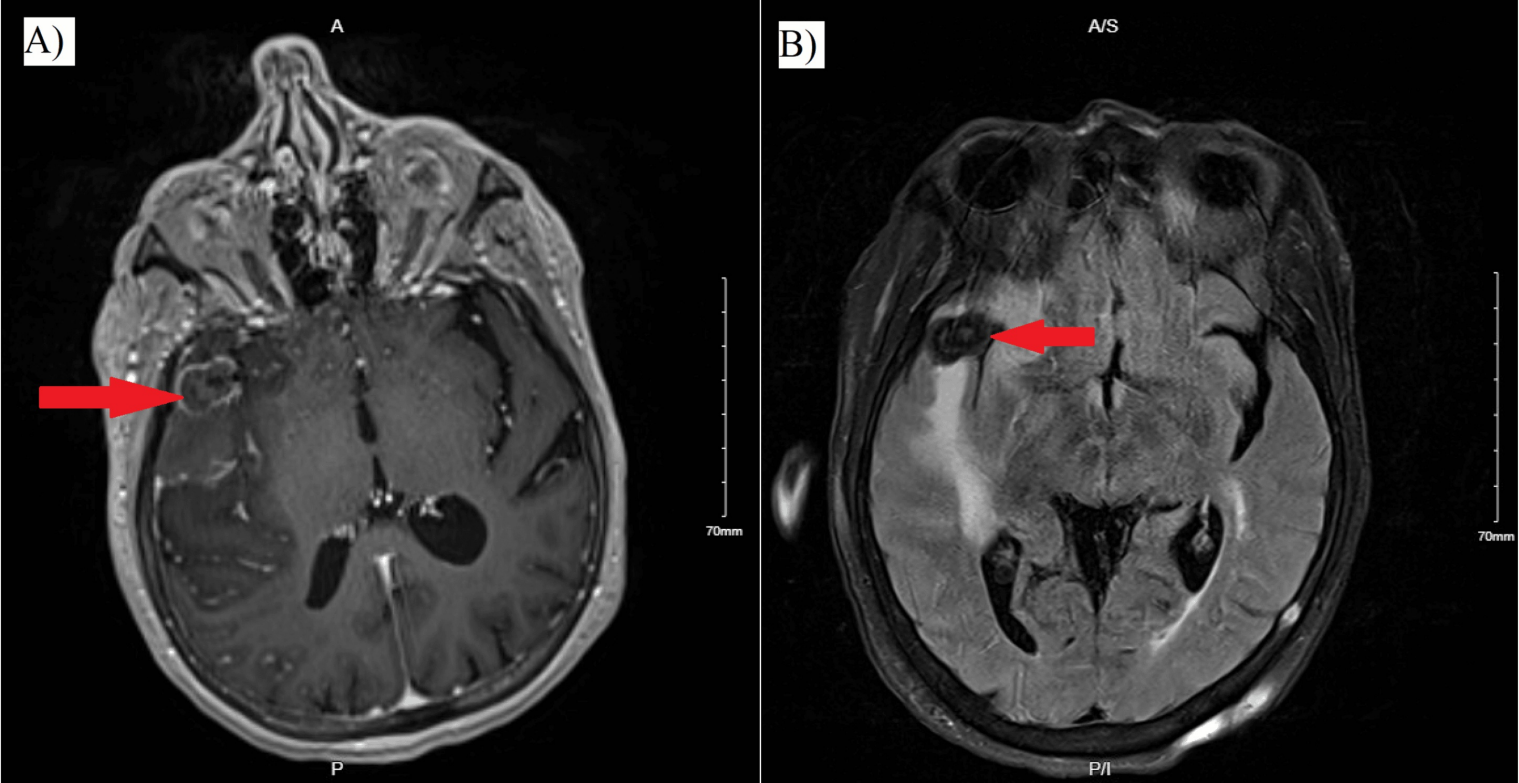
(A) Axial T1-weighted MRI showing a hypointense, peripherally enhancing large thrombosed right MCA bifurcation aneurysm, causing approximately 3 mm of leftward midline shift. (B) GRE sequence MRI demonstrating high signal intensity corresponding to the thrombosed aneurysm. GRE: gradient recalled echo; MCA: middle cerebral artery

**Figure 2:**
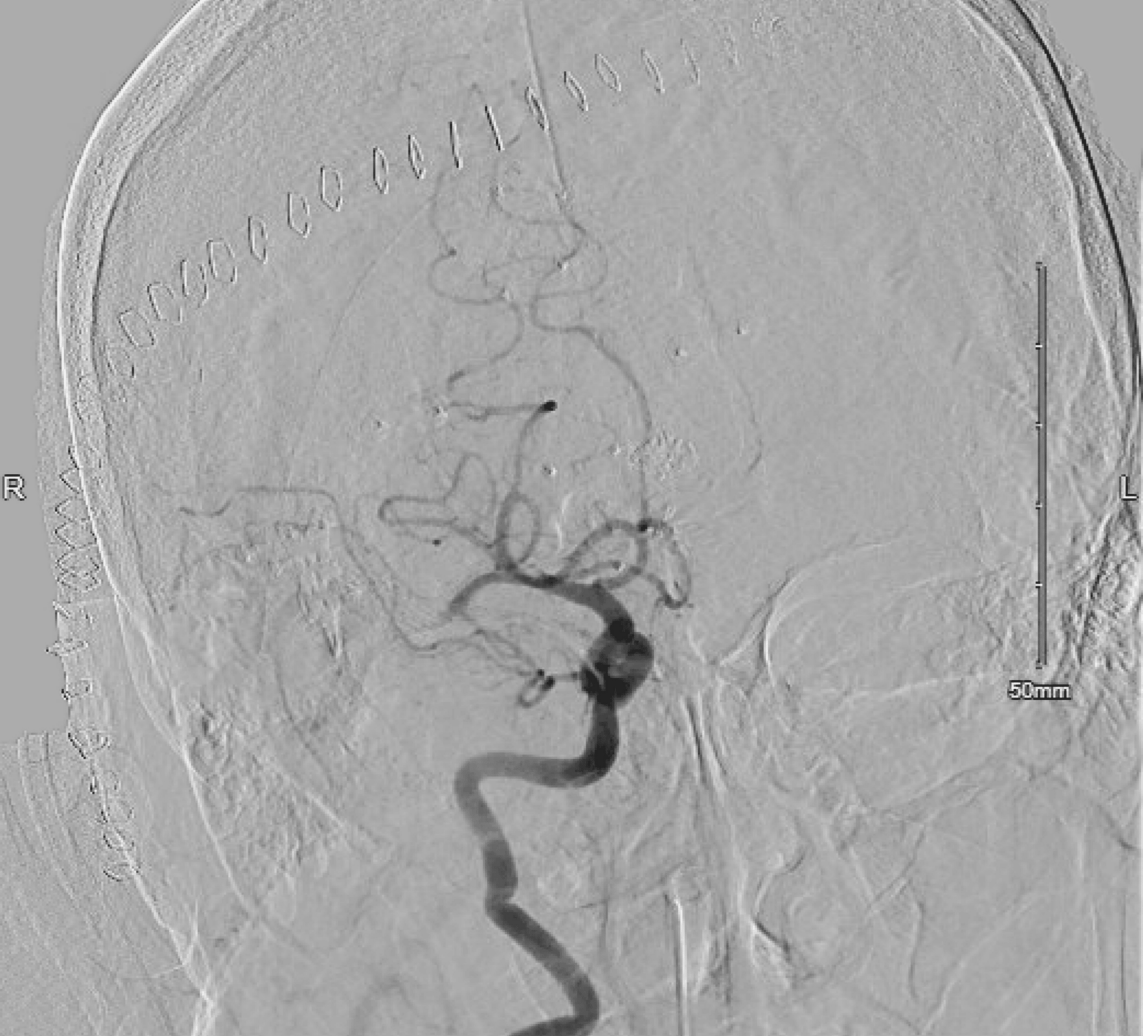
Right anteroposterior ICA digital subtraction angiography revealing M1 segment occlusion and absence of opacification in the thrombosed, large right MCA bifurcation aneurysm ICA: internal carotid artery; MCA: middle cerebral artery

**Figure 3:**
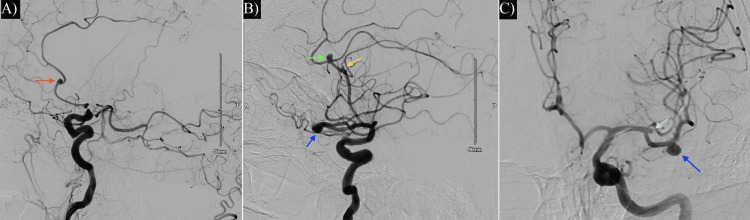
(A) Preoperative right ICA digital subtraction angiography showing a right ACA pericallosal artery aneurysm (red arrow) and lack of opacification in the large thrombosed right MCA bifurcation aneurysm. (B) Preoperative left ICA digital subtraction angiography identifying a left ACA pericallosal artery aneurysm (yellow arrow), a callosomarginal artery aneurysm (green arrow), and an M2 inferior trunk aneurysm (dark blue arrow). (C) Left ICA anteroposterior view displaying the M2 inferior trunk aneurysm (dark blue arrow) and a small left MCA bifurcation aneurysm (light blue arrow). ACA: anterior cerebral artery; ICA: internal carotid artery; MCA: middle cerebral artery

**Figure 4:**
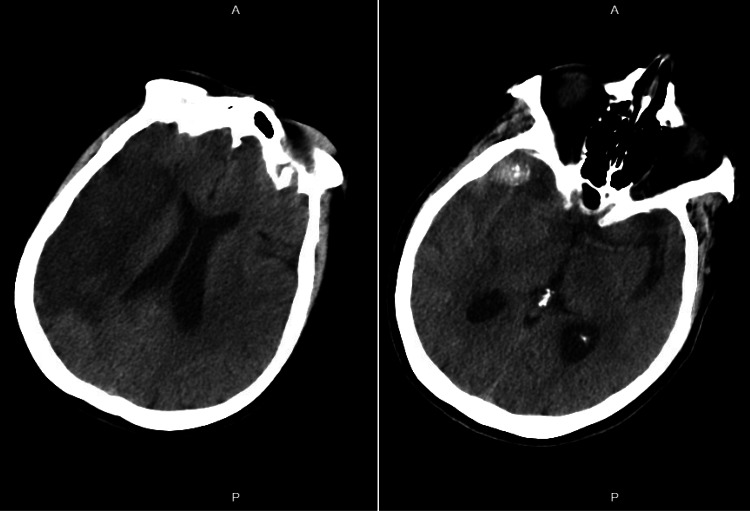
Axial CT demonstrating a hyperdense, calcified, large thrombosed MCA bifurcation aneurysm, associated with infarction and extensive cytotoxic edema in the MCA territory MCA: middle cerebral artery

**Figure 5:**
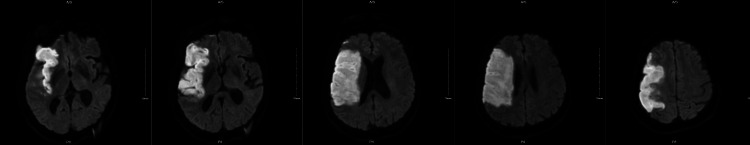
DWI sequence showing stroke in MCA territory DWI: diffusion-weighted imaging; MCA: middle cerebral artery

CT angiography and MRI demonstrated a large thrombosed right MCA bifurcation aneurysm measuring 16 mm in height by 19 mm in width, with occlusion of the M1 segment of the right MCA (Figure 1, Figure 2). This case was unique because the large thrombosed MCA bifurcation aneurysm showed complete nonopacification on right internal carotid artery (ICA) angiography, mimicking a typical M1 occlusion. Most thrombosed aneurysms, particularly large ones, partially opacify on angiography, making this presentation a notable diagnostic challenge. Further evaluation with diagnostic angiography revealed a right anterior cerebral artery (ACA) mid-A2 segment pericallosal artery aneurysm measuring 4.8 mm by 3.5 mm with a 2.7-mm neck; a bilobed left ACA proximal A3 segment pericallosal artery aneurysm measuring 3.0 mm by 5.0 mm; a left ACA callosomarginal artery aneurysm measuring 3.1 mm by 4.2 mm with a 3.1-mm neck; a wide-necked left MCA proximal M2 inferior trunk aneurysm measuring 5.5 mm by 6.6 mm with a 4.1-mm neck; and a broad-based, small left MCA bifurcation aneurysm measuring 2.8 mm by 2.5 mm (Figure 3). Additionally, moderate stenosis was noted at the left proximal cervical ICA distal carotid bulb, with 52% narrowing (3.1 mm at stenosis, 6.4 mm at mid-bulb), and the left external carotid artery origin was occluded (Figure 4, Figure 5).

**Figure 6:**
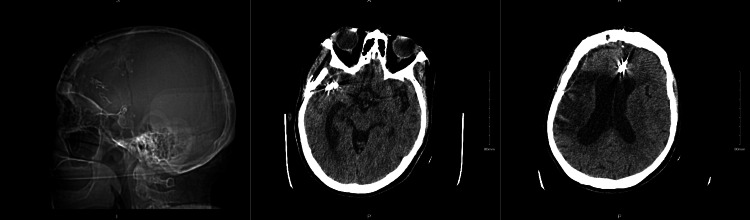
Postoperative CT scan demonstrating placement of vascular clips at the large thrombosed right MCA bifurcation aneurysm and at ACA aneurysms ACA: anterior cerebral artery; MCA: middle cerebral artery

The patient underwent a right pterional craniotomy for resection of the large, thrombosed right MCA bifurcation aneurysm. The decision to proceed with direct surgery was made to prevent further clot propagation and reduce stroke risk, as well as to secure the aneurysm. During microdissection, three MCA M2 vessel origins were identified, all thrombosed and arising from the aneurysm. The thrombosed bifurcation aneurysm began distal to the right MCA M1 segment, which entered the thrombosed aneurysm neck. A straight miniature clip was placed across each of the three MCA M2 vessel origins, and each vessel was transected at the aneurysm base. Both the right MCA M1 segment entering the thrombosed aneurysm neck and the distal M1 segment (distal to the anterior temporal artery origin) were thrombosed. The thrombosed distal M1 segment was closed with a 7-mm straight clip and transected at the aneurysm neck to allow en bloc resection of the completely thrombosed large right MCA bifurcation aneurysm. The resected aneurysm was sent to pathology, which revealed a laminated brown thrombus within the aneurysmal vessel lumen. In the same operation, a bifrontal parasagittal craniotomy was performed for clipping the incidental ACA aneurysms. No intraoperative complications were observed (Figure 6).

The postoperative angiogram showed persistent occlusion of the right MCA M1 segment, as expected. No new stroke territories were identified. The patient was discharged home to family on postoperative day 17. No further ischemic complications occurred, and her functional status was assessed as 4 on the Modified Rankin Scale.

## Discussion

Thrombosed large aneurysms should be recognized as distinctly precarious vascular lesions due to their increased risk of growth, recanalization, rupture, mass effect-related compression, and ischemic stroke [9,14]. Even in cases of complete occlusion, these aneurysms are not necessarily stable. Long-term recanalization rates range from 20% to 40%, and recanalization may lead to rapid aneurysm enlargement and eventual rupture [14]. While limited data exist on the long-term prognosis of untreated, occluded, thrombosed large aneurysms, studies on untreated very large or giant aneurysms report two-year survival rates as low as 20%, suggesting that such lesions may remain unstable and at risk for rupture even when thrombosed [15].

Most thrombosed aneurysms, particularly large ones, exhibit unfavorable characteristics and have historically been considered either untreatable or associated with high risks of poor clinical outcomes [16]. Distal thromboembolism is relatively uncommon, occurring in approximately 5-8% of cases, while acute parent artery occlusion leading to catastrophic ischemic stroke remains an exceedingly rare complication of large intracranial aneurysms [9].

Cerebral infarction can be the initial manifestation of a thrombosed intracranial aneurysm, especially in those classified as large. Several mechanisms have been proposed to explain this phenomenon, including intraluminal thrombus extension into the parent artery causing direct vascular occlusion; distal embolization of thrombotic material; and mass effect from the aneurysm compressing adjacent vessels, compromising cerebral perfusion [17]. Contributing factors to thrombus formation within aneurysms include a large dome-to-neck ratio, intrasaccular flow stasis, endothelial damage, and intramural hemorrhage [18].

The present case describes the rare occurrence of a parent M1 and multiple outflow M2 occlusions secondary to a thrombosed large MCA aneurysm, leading to a significant ischemic stroke. Given the complete thrombosis of the MCA bifurcation aneurysm and occlusion of the M1 segment, it is plausible that intrasaccular thrombus extended retrogradely into M1, resulting in direct occlusion. However, the possibility of distal embolization from clot fragmentation also cannot be excluded, as it may have contributed to more distal ischemia. Notably, the patient had multiple modifiable risk factors for cerebral aneurysm formation, including smoking, alcohol use, and hypertension [19]. Both smoking and hypertension individually confer an odds ratio of 3.0 for unruptured intracranial aneurysms; when combined, the odds ratio rises significantly to 8.3 [20]. The patient also had a history of hyperlipidemia, which is associated with a 41% increased likelihood of developing intracranial aneurysms [21].

Treatment options for large aneurysms include endovascular coiling, clipping, trapping, or reconstruction following thrombectomy [13]. Despite technological advances, large intracranial aneurysms remain highly complex, with perioperative morbidity and mortality rates ranging from 20% to 50% for both surgical and endovascular approaches. Recurrence rates may reach up to 20.7% [12]. Nevertheless, surgery remains a primary treatment modality, with conventional clipping yielding favorable outcomes in approximately one-third of cases [9]. In many instances, treatment of large lesions requires resection of the aneurysm.

In this case, the patient underwent craniotomy for resection of the thrombosed right MCA bifurcation aneurysm, along with surgical clipping of the left ACA A3 segment pericallosal aneurysm, the left ACA A3 callosomarginal artery aneurysm, and the right ACA A2 segment pericallosal aneurysm. Prior reports have described ischemic strokes resulting from spontaneous thrombosis of unruptured intracranial aneurysms in the MCA territory (Table 1). Fomenko and Kaufmann reported a case managed conservatively with aspirin and clopidogrel [7]. Arauz et al. presented three cases treated medically with aspirin, atorvastatin, and enoxaparin for deep vein thrombosis prophylaxis; only one patient underwent delayed aneurysm clipping [10]. In contrast, Kim et al. described two surgically treated cases using superficial temporal artery (STA)-to-MCA bypass and aneurysm trapping [9]. Khoa et al. reported a case managed by observation alone due to late presentation and complete aneurysmal thrombosis [11]. Sack et al. detailed a large fusiform MCA aneurysm initially treated with overlapping flow diverters and a stent; however, due to in-stent thrombosis and worsening mass effect, the patient ultimately required en bloc surgical resection and indirect STA bypass [12]. These diverse strategies underscore the lack of standardized treatment guidelines, necessitating individualized management based on clinical and radiological factors.

**Table 1:** Summary of previously reported cases of thrombosed intracranial aneurysms leading to ischemic stroke ACA: anterior cerebral artery; CTA: CT angiography; DSA: digital subtraction angiography; DWI: diffusion-weighted imaging; MCA: middle cerebral artery; PED: pipeline embolization device; PWI: perfusion-weighted imaging; STA: superficial temporal artery

Reference	Case	Clinical manifestations	Diagnosis	Stroke location	Treatment
Fomenko and Kaufmann (2016) [7]	1	Global aphasia, hemiparesis	CTA/MRI	MCA territory	Acetylsalicylic acid, clopidogrel
Brownlee et al. (1995) [8]	1	Hemiparesis	CT/MRI/DSA	ACA and MCA territories	Not documented
Kim et al. (2020) [9]	1	Hemiparesis	CTA/MRI/DSA/DWI/PWI	MCA territory	STA-MCA bypass
	2	Dysarthria, hemiparesis	CTA/MRI/DSA/DWI/PWI	MCA territory	STA-MCA bypass
Arauz et al. (2016) [10]	1	Ataxia, hemiparesis	MRI/DSA	Basilar artery	Aspirin, atorvastatin, enoxaparin
	2	Cortical blindness, hemiparesis, somnolence	MRI/DSA	Basilar artery and MCA (M1)	Aspirin, atorvastatin, enoxaparin
	3	Aphasia, hemiparesis	MRI/DSA	MCA territory	Aspirin, atorvastatin, enoxaparin; delayed aneurysm clipping (one month later)
Khoa et al. (2024) [11]	1	Dysarthria, hemiparesis	CT/MRI-DWI/DSA	MCA territory	Observation only; no treatment due to late presentation
Sack et al. (2017) [12]	1	Aphasia, hemiparesis	CTA/MRI/DSA	MCA territory	Flow diversion (PED + stent); surgical resection; indirect STA bypass

Surgical clipping remains a common treatment for intracranial aneurysms, especially saccular and anterior circulation aneurysms. While clipping offers a durable solution with a low recurrence rate, aneurysmal regrowth occurs at an annual rate of 0.26-0.53%, necessitating long-term monitoring [22]. However, it can be challenging in complex cases such as wide-necked, fusiform, or deep-seated aneurysms [13]. Large intracranial aneurysms carry a significant risk of rupture, with mortality rates ranging from 65% to 100% within one to five years after rupture [16].

## Conclusions

Large, thrombosed aneurysms present substantial diagnostic and treatment challenges due to their instability and high risk of ischemic stroke. This case underscores the importance of considering aneurysmal thrombosis in the differential diagnosis of ischemic stroke. Craniotomy for resection of a large, thrombosed right MCA bifurcation aneurysm is an effective treatment option for managing thrombosed aneurysms with associated vessel occlusion.
